# Factors predicting readmission in patients with COVID-19

**DOI:** 10.1186/s13104-021-05782-7

**Published:** 2021-09-26

**Authors:** Mohammad Nematshahi, Davood Soroosh, Mahboubeh Neamatshahi, Fahimeh Attarian, Faeze Rahimi

**Affiliations:** 1 Department of Anesthesiology, School of Medicine, Sabzevar University of Medical Science, Sabzevar, Iran; 2 Clinical Research Development Unit, Hospital Research Development Committee, Vasei Educational Hospital, Sabzevar University of Medical Sciences, TohidShar BLV, 9617747431 Sabzevar, Razavi Khorasan Iran; 3 Community Medicine Department, Sabzevar University of Medical Sciences, Sabzevar, Iran; 4 Department of Public Health, School of Health, Torbat Heydariyeh of Medical Sciences, Torbat Heydariyeh , Iran

**Keywords:** COVID-19, Readmission, SARS-CoV-2, Iran, Prediction

## Abstract

**Objective:**

COVID-19 has been introduced by the World Health Organization as a health emergency worldwide. Up to 9% of the patients with COVID-19 may be readmitted by 2 months after discharge. This study aimed to estimate the readmission rate and identify main risk factors for readmission in these patients. In this prospective study, 416 discharged COVID patients followed up with a minimum 1 month and the readmission rate was recorded. Evaluated characteristics included time of readmission, age and sex, main symptoms of disease, result of computed tomography scan, reverse transcription polymerase chain reaction test and treatment modalities.

**Results:**

Regarding readmission, 51 patients of 416 discharged patients, was readmitted during the study period. The rate of readmission for 30 and 60 days after discharge was 7.6% and 8.1%, respectively. The median age of the readmitted patients was 67 years (IQR: 53–78). About 65% of readmitted patients had underlying disease. The most significant factor in readmission rate was related to the site of lung involvement (OR > 4). Age over 60 years, underlying disease especially diabetes (OR = 3.43), high creatinine level (≥ to 1.2 mg/dl) (OR = 2.15) were the most important predictors of readmission.

**Supplementary Information:**

The online version contains supplementary material available at 10.1186/s13104-021-05782-7.

## Introduction

Coronavirus Disease 2019 (COVID-19) was declared by the World Health Organization as a global health emergency on January 30, 2020 [1, 2]. SARS-CoV-2 transmission rate is high but most people recover following COVID-19 like Severe Acute Respiratory Syndrome (SARS), and Middle East Respiratory Syndrome (MERS) [3, 4]. However in 5% of cases, hospitalization is essential to keep on the treatment process [5, 6], in other side the clear guidelines for the management of the patients with COVID-19 and the time of discharge have not yet been determined based on clinical evidence [5, 7]. According to the Centers for Disease Control and Prevention (CDC), up to 9% of the patients with COVID-19 may be readmitted within 2 months of discharge [8, 9]. Mostly male, old age and the presence of underlying disease or history of malignancy are associated with poor prognosis and readmission in this group of patients [10–12]. Comorbidities or heart disease were associated with higher odds of readmission in these patients [13–16]. Increment of readmission and ignoring related risk factors could lead to serious form of diseases and mortality based on the experience of previous respiratory pandemics [3, 17–19]. Not only COIVD-19 causes multi-organ failure, but also drugs used to treat COVID-19 cause complications, therefore follow up is essential to complete the treatment course [20–22]. Paying attention to the clinical aspects of readmission in these patients is fundamental to make better decision in follow-up [10, 11, 23, 24]. This study conducted to estimate the rate of readmission and identify main risk factors for readmission in the patients with COVID-19.

## Main text

### Methods and materials

#### Study setting & design

We performed prospective study on patients admitted with a diagnosis of COVID-19 in Vasei hospital, Khorasan Razavi province, Iran, between March 1, 2019 and May 20, 2020. The protocol of study was approved by ethics committee of Sabzevar University of Medical Sciences (IR.MEDSAB.REC.1399.004) and informed consent form was obtained from all patients. The data were analyzed through STATA.

### Study population

Study population was a cohort of 506 patients admitted with definite or clinical diagnosis of COVID-19. Diagnosis of COVID-19 was ascertained by a positive result of polymerase chain reaction (PCR) and imaging finding. In this study, 92 patients who died during hospitalization were excluded from the study and 416 patients were discharged and followed up for at least 6 months.

### Data collection

Clinical and demographic variables were included age, sex, symptoms, biochemical findings, CT scan and RT-PCR test results and underlying disease, pregnancy, and readmission rate, extracted from health information system (HIS). Comorbidities were measured using ICD-10 and categorized in three main groups diabetes, hypertension and other disease. Main symptoms and clinical variables that assessed in these patients were dry cough, fever and Spo2. Also, we assessed D-dimer (no/ yes) and level of creatinine (mg/dl). Additionally, some of other variable were recorded such as length of stay at hospital and use of mechanical ventilation. Moreover status of ground glass opacity and site of lesions was assessed in patient whit abnormal result through lung CT scans.

### Main measurements

Readmission was evaluated as a primary outcome at a time interval of 3 to 180 days after discharge; secondary outcome was death in readmitted cases.

## Results

### Readmission time of patients

In this study, 416 discharged patients were followed up, the mean follow-up was 61 ± 11.2 days. Fifty-one of the patients were readmitted, which 13 patients (25%) 1 week after discharge and 19 patients up to 30 days after discharge and 2 patients up to 60 days after discharge and the rest of the patients (n = 17) up to 40 weeks after discharge were readmitted. Readmission rate in 1 week, 30 days, 60 days and up to 40 weeks after discharge were 3.1%, 7.6%, 8.1% and 12.25%, respectively.

### Characteristics of the patients

The mean age of the readmitted patients (n = 51) 65 ± 15.42 years. The mean age of the recovered patients was 56 ± 18.2 years (p < 0.001). The results showed that there was a significant relationship between underlying disease and readmission (p < 0.001). Two thirds of readmitted patients had a history of negative PCR results, although 84.3% of the readmitted patients had abnormal chest imaging results in the first hospitalization (Table 1).

**Table 1:** Characteristics of the recovered and readmitted patient

Variable	Total number%	Non-readmitted, 365 (87.7)		Readmitted, 51 (12.2)		P-value
		N	%	N	%	
Sex (n = 416)						
Male	228 (54.0)	203	55.6	25	49.0	0.78*
Female	188 (45.0)	162	44.4	26	51.0	
PCR (n = 368)						
Negative	227 (61.6)	169	61.2	31	64.6	0.19*
Positive	141 (38.4)	124	38.8	17	35.4	
Comorbidity (n = 416)						
No	224 (53.8)	206	56.4	18	35.3	< 0.001**
Diabetes	42 (10.1)	29	8.0	13	25.5	
Hypertension	61 (14.7)	55	15.0	6	11.7	
Others	83 (20.0)	69	18.9	14	27.4	
Pregnancy	6 (01.4)	6	1.64	0	0	
D-dimer (n = 416)						
No	85 (19.7)	78	21.4	7	13.7	0.20*
Yes	331 (80.3)	287	78.6	44	86.3	
Age (year)						
≤ 60	226 (54.3)	205	56.2	21	42.1	0.04*
> 60	190 (45.7)	160	43.8	30	58.9	
Quantitative variables	N	N	Mean (CI %95)	N	Mean (CI %95)	P value ***
SPO2	407	356	89.3 ( 88.4, 90.2)	51	88.2 ( 86.3, 90.1)	0.40
Creatinine (mg/dl)	372	326	1.1 (1.1, 1.2)	46	1.5 (1.2, 1.9)	< 0.001
Hospitalization (day)	415	364	5.2 (4.7, 5.7)	51	7.0 (5.5, 8.5)	< 0.001
ICU (day)	52	43	4 (2.75, 5.24)	9	3.4 (0.36, 7.25)	0.72

^*^Pearson χ2, ** Fisher's exact, *** t-test

However, 84.3% of the readmitted patients had a history of abnormal CT scan presented in detail in Table 2.

**Table 2:** Comparison of readmission time of patients based on CT scan

Variable	Total n%	Readmission						Log rank test
		3–7 days		8–30 days		31–60 days		
		N	Failure function	N	Failure function	N	Failure function	
History of CT result (n = 51)								
Normal	8 (15.7)	1	0.12	2	0.25	3	0.37	P = 0.04
Abnormal	43 (84.3)	12	0.27	30	0.69	31	0.72	
Ground Glass Opacity (n = 43)								
No	6 (13.95)	2	0.33	3	0.50	3	0.50	P = 0.65
Yes	37 (86.05)	10	0.27	27	0.72	28	0.75	
Site of lesions (n = 43)								
Multiple lobes	4 (9.30)	3	0.75	4	1.0			P = 0.04
Peripheral	20 (46.51)	6	0.30	15	0.75	16	0.80	
Lung bases	14 (32.56)	3	0.21	10	0.71	10	0.71	
Peribronchovascular distribution	2 (4.65)	–	–	–	–	–	–	
Central	3 (6.96)	0	0	1	0.33	1	0.33	
Bilateral								
No	12 (27.91)	4	0.33	4	0.33	4	0.33	P = 0.002
Yes	31 (72.09)	8	0.25	26	0.83	27	0.87	
Pleural effusion								
No	39 (90.7)	10	0.25	26	0.66	27	0.69	P = 0.38
Yes	4 (9.3)	2	0.50	4	1.0			

### Risk factor for readmission

To estimate and predict risk factor, regression models are helpful [25]. Risk of readmission for each of the related factors was estimated using regression model [26]. To find relationship between demographic, clinical, biochemical and therapeutic characteristics and dependent variable (readmission), regression models are used. The distribution of the data was normal checking with Kolmogorov–Smirnov test. After controlling the effect of other variables, the odds ratio of readmission in the patients with abnormal creatinine level and diabetes was 2.15 and 3.43, respectively (Table 3).

**Table 3:** Relationship between characteristics and readmission of COVID-19 patients

Adjusted OR (95% CI)	Crude OR (95% CI)	Variable
Age		
≥ 60	1	1
> 60	1.83 (1.00, 3.31)*	1.12 (0.52, 2.47)
Sex		
Male	1	–
Female	1.30 (0.72, 2.34)	–
Dry cough		
No	1	–
Yes	0.68 (0.37, 1.23)	–
Fever		
No	1	–
Yes	0.62 (0.34, 1.12)	–
Comorbidity		
No	1	1
DM	5.27 (2.34, 11.89)*	3.43 (1.13, 8.37)*
HTN	1.28 (0.48, 3.39)	0.72 (0.21, 2.47)
Others	2.38(1.13, 5.05)*	1.24 (0.46, 3.27)
D-dimer (ng/ml)		
No	1	–
Yes	1.70 (0.74, 3.94)	–
PCR		
No	1	–
Yes	0.86 (0.46, 1.63)	–
Creatinine, mg/dL		
< 1.2	1	1
≥ 1.2	2.84 (1.48, 5.43)*	2.15 (1.00, 4.59)*
Duration of hospitalization		
< 6	1	1
≥ 6	1.93 (1.06, 3.51)*	2.00(0.94, 4.27)
Admission to ICU		
No	1	–
Yes	1.60(0.73, 3.52)	–
CT result		
Normal	1	–
Abnormal	1.48 (0.67, 3.28)	–
Site of lesion(s)		
Multiple lobes	1	1
Peripheral	3.36 (1.10, 10.19)*	2.40 (0.75, 7.67)
Lung bases	4.41 (1.38, 14.05)*	4.16 (1.23, 14.05)*
Peribronchovascular distribution	4.55 (0.72, 28.4)	4.43 (0.65, 30.51)
Central	20.5 (3.10, 135.5)*	6.15 (0.66, 56.93)

## Discussion

In this study the rate of readmission with the same interval was consistent with other studies, in which readmission in the first week after discharge was between 2 and 4% [23, 24, 27] and was reported 10% for 2 months after discharge [8]. In the follow-up of 279 discharged patients in Rhode Island of the United States, 30 days after discharge, readmission rate was reported 6.7% [10]. In Turkish study, 7.1% of discharged patients were readmitted [12].The readmission rate 2 months after discharge was 9% in the United States [8]. Donnelly et al. reported readmission rate up to 20% 2 months after discharge in the United States [28]. In another study, 10.3% of the discharged patients were readmitted to hospital 80 days after discharge [29]. However, in different findings in the Korean population, the readmission rate after discharge was 4.3% [11] and in Spanish population, the readmission rate was 4.4% up to 3 weeks after discharge [9].

The median length of stay at hospitals for the first time was 4 days similar to the findings of previous studies in Iran [30, 31]. A review of 52 studies estimated the median length of stay at hospitals up to 5 days [32] similar to the median length of stay in New York performed on 5700 patients [23]. Length of stay was very different in Korean hospitals (17 days) [11]. Also, in the present study, the median length of stay for readmitted and non-readmitted patients was 5 days (IQR: 3–9) and 4 days (IQR: 3–6), respectively, while in the Turkish study, the median length of stay was 4 days and 3 days, respectively [12].

The median length of stay in the Spanish readmitted patients with COVID-19 was lower than other patients (6 days vs. 9 days) [9]. Hospitalization of the patients has some conditions which include the capacity of the medical system, the quality of care and the demand for hospital beds in the pandemic period [12, 17, 33]. Different quality of post-discharge care can also have main role in the completion of the treatment process [24, 29].

Wu and McGoogan [34] and Richardson et al. [23] revealed that the odds ratio of readmission in elderly patients was higher than in other age groups. Underlying diseases, especially hypertension and diabetes, have also been confirmed in other COVID-19 studies [9, 10, 12, 24]. Aging reduces the response of immune cells to SARS-CoV-2; consequently the virus may be able to stay in the body longer. Complications in readmitted COVID-19 patients are more in elderly people [35], however these two factors may be strong predictors for readmission in the hospitalized patients [36]. High creatinine level in the hospitalized patients was another predictor for readmission in COVID-19 patients, so that the odds ratio of readmission for a creatinine level greater than 1.2 mg/dl was 2.15. Findings of the previous studies on other diseases also consider creatinine level as an effective factor for readmission of COVID-19 patients [37–39].

This finding was consistent with the findings of previous studies that introduced abnormal chest imaging results as a predictor of readmission [11, 40], so chest imaging is a suitable tool for managing COVID-19 patients after discharge [41–43]. Also, the possibility of a false negative result in PCR test was reported in previous studies [41]. At the end, the key point to indirectly avoid readmission is fully vaccination of the patients after recovery from the COVID-19 through approved vaccine as soon as possible [44, 45].

## Conclusion

According to the results, age over 60 years, underlying disease especially diabetes, high creatinine level, duration of first-time hospitalization and lung involvement were the most important predictors of readmission in the patients with COVID-19. The site of lung involvement was had very crucial role in readmission with odds ratio of 4.5 for peribronchovascular distribution or central involvement and odds ratio of 4.16 for basal lung involvement. The readmission rate was 3.1% for 1 week after discharge, 7.6% for 1 month after discharge, and 8.6% for 2 months after discharge.

## Limitation

One of the main limitations in the present study was small sample size which makes it hard to generalize the obtained results.

## Supplementary Information

**Additional file 1.** Raw data for possible analysis and review studies.

## Data Availability

The datasets analysed during the current study are available from the corresponding author on reasonable request (Additional file 1).

## Abbreviations

COVID-19: Coronavirus Disease 2019
WHO: World Health Organization
CDC: Centers for Disease Control and Prevention
PCR: Polymerase Chain Reaction
ICUs: Intensive Care Units

## References

[1] Audrey S, Procter S (2015). Employers’ views of promoting walking to work: a qualitative study. Int J Behav Nutr Phys Act.

[2] Schøsler L, Christensen LA, Hvas CL (2016). Symptoms and findings in adult-onset celiac disease in a historical Danish patient cohort. Scand J Gastroenterol.

[3] Ganji R, Moghbeli M, Sadeghi R, Bayat G, Ganji A (2019). Prevalence of osteoporosis and osteopenia in men and premenopausal women with celiac disease: a systematic review. Nutr J..

[4] Daneshfar M, Dadashzadeh N, Ahmadpour M, RagatiHaghi H, Rahmani V, Forouzesh M (2021). Lessons of mortality following COVID-19 epidemic in the United States especially in the geriatrics. J Nephropharmacol..

[5] Yeo I, Baek S, Kim J, Elshakh H, Voronina A, Lou MS (2021). Assessment of thirty-day readmission rate, timing, causes and predictors after hospitalization with COVID-19. J Intern Med.

[6] Zanchetta MB, Longobardi V, Bai JC (2016). Bone and celiac disease. Curr Osteoporos Rep..

[7] Micic D, Rao VL, Semrad CE (2020). Celiac disease and its role in the development of metabolic bone disease. J Clin Densitom..

[8] Bergamaschi G, Markopoulos K, Albertini R, Di Sabatino A, Biagi F, Ciccocioppo R (2008). Anemia of chronic disease and defective erythropoietin production in patients with celiac disease. Haematologica.

[9] Parra LM, Cantero M, Morrás I, Vallejo A, Diego I, Jiménez-Tejero E (2020). Hospital readmissions of discharged patients with COVID-19. Int J General Med.

[10] Atalla E, Kalligeros M, Giampaolo G, Mylona EK, Shehadeh F, Mylonakis E (2021). Readmissions among patients with COVID-19. Int J Clin Pract..

[11] Jeon W-H, Seon JY, Park S-Y, Oh I-H (2020). Analysis of risk factors on readmission cases of COVID-19 in the Republic of Korea: using nationwide health claims data. Int J Environ Res Public Health.

[12] UyaroĞlu OA, BaŞaran NÇ, ÖziŞik L, Dİzman GT, EroĞlu İ, Şahİn TK, TaŞ Z, İnkaya AÇ, TanriÖver MD, Metan G, GÜven GS, Ünal S. Thirty-day readmission rate of COVID-19 patients discharged from a tertiary care university hospital in Turkey: an observational, single-center study. Int J Qual Health Care. 2021;33:144. 10.1093/intqhc/mzaa144 PMC7665548 33104780

[13] Verna EC, Landis C, Brown RS, Mospan AR, Crawford JM, Hildebrand JS (2021). Factors associated with readmission in the US following hospitalization with COVID-19. Clin Infect Dis.

[14] Ramos-Martínez A, Parra-Ramírez LM, Morrás I, Carnevali M, Jiménez-Ibañez L, Rubio-Rivas M (2021). Frequency, risk factors, and outcomes of hospital readmissions of COVID-19 patients. Sci Rep.

[15] Ye S, Hiura G, Fleck E, Garcia A, Geleris J, Lee P (2021). Hospital readmissions after implementation of a discharge care program for patients with COVID-19 illness. J Gen Intern Med.

[16] Donnelly JP, Wang XQ, Iwashyna TJ, Prescott HC (2021). Readmission and death after initial hospital discharge among patients with COVID-19 in a large multihospital system. JAMA.

[17] Battershill PM (2006). Influenza pandemic planning for cancer patients. Curr Oncol.

[18] Dafer RM, Osteraas ND, Biller J. Acute stroke care in the coronavirus disease 2019 pandemic. Elsevier; 2020. 10.1016/j.jstrokecerebrovasdis.2020.104881 PMC7164903 32334918

[19] McBride KE, Brown KG, Fisher OM, Steffens D, Yeo DA, Koh CE (2020). Impact of the COVID-19 pandemic on surgical services: early experiences at a nominated COVID-19 centre. ANZ J Surg.

[20] Rahimi MM, Jahantabi E, Lotfi B, Forouzesh M, Valizadeh R, Farshid S (2021). Renal and liver injury following the treatment of COVID-19 by remdesivir. J Nephropathol..

[21] Lotfi B, Farshid S, Dadashzadeh N, Valizadeh R, Rahimi MM (2020). Is coronavirus disease 2019 (COVID-19) associated with renal involvement? A review of century infection. Jundishapur J Microbiol..

[22] Barzegar A, Ghadipasha M, Rezaei N, Forouzesh M, Valizadeh R (2021). New hope for treatment of respiratory involvement following COVID-19 by bromhexine. J Nephropharmacol..

[23] Richardson S, Hirsch JS, Narasimhan M, Crawford JM, McGinn T, Davidson KW (2020). Presenting characteristics, comorbidities, and outcomes among 5700 patients hospitalized with COVID-19 in the New York City area. JAMA.

[24] Somani S, Richter F, Fuster V, De Freitas J, Naik N, Sigel K, et al. Characterization of patients who return to hospital following discharge from hospitalization for COVID-19. medRxiv. 2020. 10.1007/s11606-020-06120-6 PMC7437962 32815060

[25] Inan TT, Samia MB, Tulin IT, Islam MN. A decision support model to predict icu readmission through data mining approach. InPACIS 2018: 218.

[26] Osman AA, Al Daajani MM, Alsahafi AJ (2020). Re-positive COVID-19 PCR test: could it be a reinfection?. N Microb N Infect.

[27] Wang X, Xu H, Jiang H, Wang L, Lu C, Wei X (2020). The clinical features and outcomes of discharged coronavirus disease 2019 patients: a prospective cohort study. QJM Int J Med..

[28] Donnelly JP, Wang XQ, Iwashyna TJ, Prescott HC (2021). Readmission and death after initial hospital discharge among patients with COVID-19 in a large multihospital system. JAMA.

[29] McCarthy CP, Murphy S, Jones-O’Connor M, Olshan DS, Khambhati JR, Rehman S (2020). Early clinical and sociodemographic experience with patients hospitalized with COVID-19 at a large American healthcare system. EClinicalMedicine..

[30] Shahriarirad R, Khodamoradi Z, Erfani A, Hosseinpour H, Ranjbar K, Emami Y (2020). Epidemiological and clinical features of 2019 novel coronavirus diseases (COVID-19) in the South of Iran. BMC Infect Dis.

[31] Toutkaboni MP, Askari E, Khalili N, Tabarsi P, Jamaati H, Velayati AA (2020). Demographics, laboratory parameters and outcomes of 1061 patients with coronavirus disease 2019: a report from Tehran, Iran. N Microb N Infect.

[32] Rees EM, Nightingale ES, Jafari Y, Waterlow NR, Clifford S, Pearson CA, et al. COVID-19 length of hospital stay: a systematic review and data synthesis. 2020. 10.1186/s12916-020-01726-3 PMC7467845 32878619

[33] Islam MN, Inan TT, Rafi S, Akter SS, Sarker IH, Islam AN (2021). A systematic review on the use of AI and ML for fighting the COVID-19 pandemic. IEEE Trans Artif Intel.

[34] Wu Z, McGoogan JM (2020). Characteristics of and important lessons from the coronavirus disease 2019 (COVID-19) outbreak in China: summary of a report of 72 314 cases from the Chinese Center for Disease Control and Prevention. JAMA.

[35] Wong J, Koh WC, Momin RN, Alikhan MF, Fadillah N, Naing L. Probable causes and risk factors for positive SARS-CoV-2 test in recovered patients: Evidence from Brunei Darussalam. medRxiv. 2020. 10.1002/jmv.26199 PMC7323238 32558947

[36] Zapatero A, Barba R, Marco J, Hinojosa J, Plaza S, Losa JE (2012). Predictive model of readmission to internal medicine wards. Eur J Intern Med.

[37] Tamhane U, Voytas J, Aboufakher R, Maddens M (2008). Do hemoglobin and creatinine clearance affect hospital readmission rates from a skilled nursing facility heart failure rehabilitation unit?. J Am Med Dir Assoc.

[38] Michtalik HJ, Yeh H-C, Campbell CY, Haq N, Park H, Clarke W (2011). Acute changes in N-terminal pro-B-type natriuretic peptide during hospitalization and risk of readmission and mortality in patients with heart failure. Am J Cardiol.

[39] Ben-Assuli O, Padman R, Leshno M, Shabtai I (2016). Analyzing hospital readmissions using creatinine results for patients with many visits. Procedia Comput Sci.

[40] Cao B, Wang Y, Wen D, Liu W, Wang J, Fan G (2020). A trial of lopinavir–ritonavir in adults hospitalized with severe Covid-19. N Engl J Med..

[41] Xiao AT, Tong YX, Zhang S (2020). False-negative of RT-PCR and prolonged nucleic acid conversion in COVID-19: rather than recurrence. J Med Virol.

[42] Ai T, Yang Z, Hou H, Zhan C, Chen C, Lv W, et al. Correlation of chest CT and RT-PCR testing in coronavirus disease 2019 (COVID-19) in China: a report of 1014 cases. Radiology. 2020; 200642. 10.1148/radiol.2020200642 PMC7233399 32101510

[43] Li Y, Xia L (2020). Coronavirus disease 2019 (COVID-19): role of chest CT in diagnosis and management. Am J Roentgenol.

[44] Thompson MG, Stenehjem E, Grannis S, Ball SW, Naleway AL, Ong TC, DeSilva MB, Natarajan K, Bozio CH, Lewis N, Dascomb K, Dixon BE, Birch RJ, Irving SA, Rao S, Kharbanda E, Han J, Reynolds S, Goddard K, Grisel N, Fadel WF, Levy ME, Ferdinands J, Fireman B, Arndorfer J, Valvi NR, Rowley EA, Patel P, Zerbo O, Griggs EP, Porter RM, Demarco M, Blanton L, Steffens A, Zhuang Y, Olson N, Barron M, Shifflett P, Schrag SJ, Verani JR, Fry A, Gaglani M, Azziz-Baumgartner E, Klein NP (2021). Effectiveness of Covid-19 vaccines in ambulatory and inpatient care settings. N Engl J Med.

[45] Ghiasi NVR, Arabsorkhi M, Hoseyni TS, Esfandiari K, Sadighpour T, Jahantigh HR (2021). Efficacy and side effects of Sputnik V, Sinopharm and AstraZeneca vaccines to stop COVID-19; a review and discussion. Immunopathologia Persa.

